# Quality of life, and quality of sleep in the working population with chronic low back pain: A 2-year follow-up

**DOI:** 10.1097/MD.0000000000045321

**Published:** 2025-10-24

**Authors:** Aleksandar Stojanov, Gordana Djordjevic, Srdjan Ljubisavljevic, Biljana Zivadinovic, Jelena Stojanov

**Affiliations:** a Clinic of Neurology, University Clinical Center Nis, Nis, Serbia; b Medical Faculty, University of Nis, Nis, Serbia; c Special Psychiatric Hospital “Gornja Toponica”, Nis, Serbia.

**Keywords:** low back pain, quality of life, quality of sleep, working population

## Abstract

Chronic low back pain is a frequent medical condition, and a major problem especially among the working population, because it influences their working ability. The research aimed to evaluate the influence of pain on quality of life (QoL), and quality of sleep (QoS) in patients suffering from chronic low back pain, during 2 years of follow-up. The study included 128 patients. Patients were tested on 4 occasions (on admission, after 6, 12, and 24 months from initial testing). Based on age, they were divided into 3 groups: I group 18 to 30 years; II group 30 to 50 years; III group 50 to 65 years. The SF36 QoL questionnaire, Pittsburgh sleep quality index (PSQI), and the Hamilton scales for the assessment of anxiety (HARS) and depression (HDRS) were used. We did not find any statistically significant correlation between worse QoL and QoS with patients’ epidemiological factors, but younger patients tend to have slightly worse scores. Elderly patients were more disabled (*P* <.01). In young individuals, SF36 scores were significantly higher (*P* <.05), and PSQI scores were lower (*P* <.01) than in the elderly. Neuropathic pain was an independent predictor of the lower SF36 scores – which indicates worse QoL (adjusted R^2^ = 0.73, *P* <.01 for the overall model) and higher PSQI scores – which indicates worse QoS (adjusted R^2^ = 0.71, *P* <.01 for the overall model). Neuropathic pain is a predictor of worse QoL and QoS. During the follow-up reduction in pain is noticed in all groups, but more pronounced in patients younger than 50.

## 1. Introduction

Low back pain is a common symptom among middle and old-age patients, but it is also not uncommon in young adulthood (population up to 30 years old).^[1,2]^ Between 15% and 40% of people who have acute low back pain will have chronic pain or frequent relapses of acute low back pain.^[3]^ Chronic low back pain (CLBP) is a consequence of complex interactions of biological, psychological, and social factors.^[4]^ Some factors are recognized as causes for the chronification of acute pain such as female gender, genetic factors, lifestyle, poor coping mechanisms, traumatic injury, and occupational hazards.^[5]^ CLBP is associated with impaired physical functioning, higher levels of subclinical anxiety, and depression.^[6]^ This can influence patients’ quality of life, sleep, and also their ability to be a productive individual in society.

The working ability of patients who are still active workers (aged between 18 and 65 years old) is lower in patients with this condition. CLBP is one of the leading causes of worldwide productivity loss as measured in years, and one of the top causes of years lived with disability.^[7]^ Costs of diagnostic and therapeutic procedures for patients with low back pain in the USA are estimated to exceed 100 billion US dollars per year.^[8]^ Two-thirds of the economic costs connected with CLBP are a consequence of indirect costs (e.g., loss of productivity).^[9]^

This research aimed to evaluate the quality of life (QoL) and sleep (QoS) in working-population patients suffering from CLBP over a period of 2 years and to determine how it is influenced by the severity and type of pain. Hypotheses were that the presence of neuropathic pain significantly influences patients’ QoL and QoS and that pain reduction ameliorates QoL and QoS over time.

## 2. Methods

The study included 128 adult patients aged 18 to 65 (65 years are retirement age for the male population in Serbia, 61 years for females) admitted to the Clinic of Neurology Nis, from January 2021 to December 2022. Retired female patients aged 61 to 65 were excluded. Clinic of Neurology Nis is a reference institution for treating patients with chronic pain conditions (excluding oncology and postsurgical pain) so the majority of patients with low back pain, from the territory of southeastern Serbia, are instructed to come to this Institution for diagnostic testing. The control group comprised 113 age- and sex-matched volunteers, recruited via emails that were sent on the addresses of volunteers who were included in previous trials implemented in our Clinic.

The study procedures were conducted with approval from the local clinical research ethics committee. All procedures were conducted in accordance with the committee’s guidelines and regulations, including the Basics of Good Clinical Practice, the Declaration of Helsinki and the Law on Health Care of the Republic of Serbia. All subjects gave their written informed consent for participating in the study.

Only patients with a chronic condition were included (pain that continues for 12 weeks or longer). CLBP was diagnosed based on the patient’s history and physical examination. Data on the epidemiological and clinical characteristics were collected. For all patients on admission, electromyography, nerve conduction study, and imaging of the low back spine were carried out. Patients were tested on 4 occasions (on admission, after 6, 12, and 24 months from initial testing). The control group was tested 1 time only, so the results from this testing are used for comparing with the results obtained in all 4 time points for patients with CLBP. Patients were divided into 3 groups based on their age: I group 18 to 30 years old (younger working adults); II group 30 to 50 years old (middle-aged working adults); III group 50 to 65 years old (elderly working population). Only patients who were treated with conservative methods were included in this research (patients who had undergone an operation were excluded). Patients were treated with: analgesic therapy, co-analgesic therapy (if neuropathic pain is present), physical therapy, muscle relaxants, topical pain reliefs, lifestyle modifications. Also, only patients who finished follow-up are taken into account for the statistical process. The sample was determined in the Open Epi 3.01 software package. The initial parameters were defined for the study power of 80% and the probability of a type I error (α) of 0.05 for 2-sided null hypothesis testing based on the estimated prevalence. Due to the possibility of attrition during the follow-up period, 10% of the sample size was added to the calculated representative sample size.

Oswestry Low Back Pain Disability Index (ODI) was used for assessing the disability of patients.^[10]^ The scores from the 10-items are summed and expressed as a percentage (0 to 100%). A 10-cm visual analogue scale with “no pain” on the left side and “worst pain” on the right side was used to measure worst pain intensity in the last week.^[11]^ Also, the PainDETECT questionnaire (PDQ) was used for assessing the neuropathic component of the pain.^[12]^ For patients who have a diagnosed neuropathic pain on their first testing, we make an insight into their clinical neurological examination, neurophysiologic tests, and imaging findings. If no abnormality was found in these tests (which can confirm the diagnosis of neuropathic pain), we exclude these patients from future study procedures, and their findings were not taken into account for statistical analyses.

For assessing life quality, a 36-item short-form survey Instrument (SF36) was used. SF36 measures 8 general health dimensions. The scores for each of the 8 domains and total score were converted to a 0 to 100 scale, with a higher score representing better health. Quality of sleep (QoS) was assessed by the Pittsburgh Sleep The obtained global Pittsburgh sleep quality index (PSQI) score is from 0 to 21, where higher scores indicate lower QoS. A total score of more than 5 indicates poor QoS.^[13]^ In addition, Hamilton scales for the assessment of anxiety (HARS) and depression (HDRS) were also used.^[14,15]^ Questionnaires were assigned to patients only after carrying out a clinical examination and collecting epidemiological data.

All data were statistically processed by IBM SPSS statistical software (version 21) for the Windows operating system. P values of <0.05 were regarded as statistically significant. Numerical data are presented as means ± standard deviation. The Mann-Whitney test was used to compare continuous variables between 2 groups, and the Kruskal-Wallis test was used to compare more than 2 groups. Correlations were assessed using Pearson correlation coefficients or Spearman correlation coefficients.

## 3. Results

**Figure 1.**
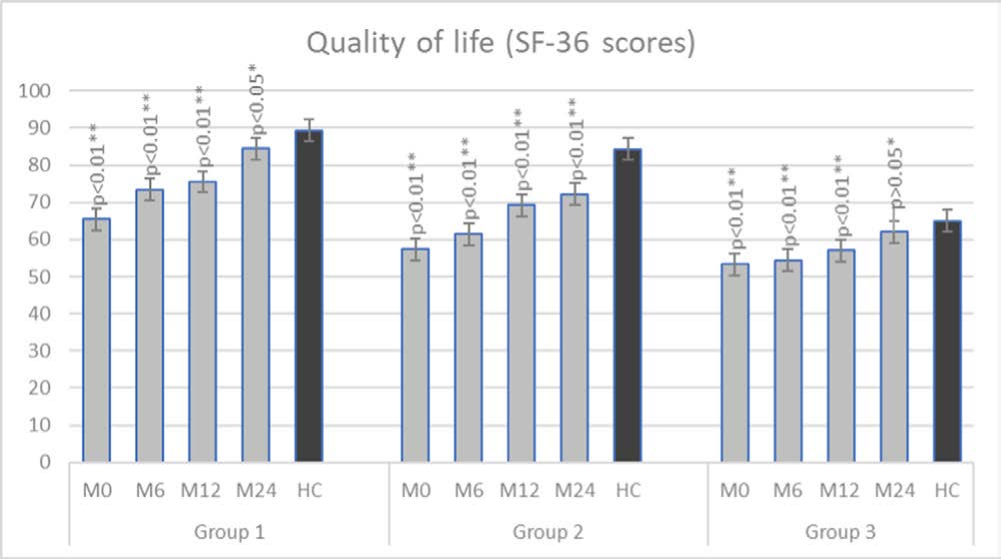
Scores on the quality of life questionnaire during the follow-up period compared to the healthy control group (M0 – on admission, M6 – month 6, M12 – month 12, M24 – month 24; HC; Group 1–18 to 30 yr old, Group 2–30 to 50 yr old, Group 3–50 to 65 yr old). HC = healthy control.

**Figure 2.**
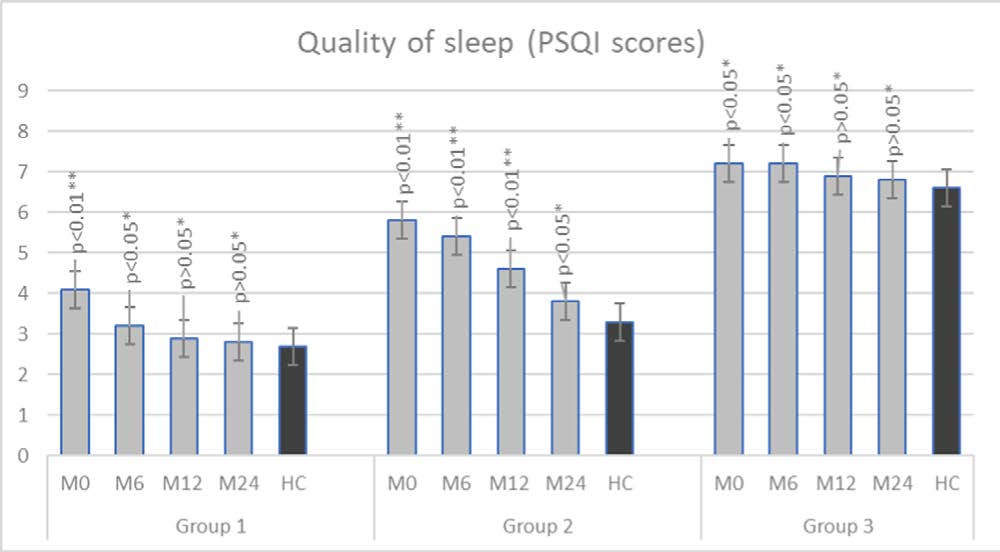
Scores on the quality of sleep questionnaire during the follow-up period compared to the healthy control group (M0 – on admission, M6 – month 6, M12 – month 12, M24 – month 24; HC; Group 1–18 to 30 years old, Group 2–30 to 50 years old, Group 3–50 to 65 years old). HC = healthy control.

Epidemiological characteristics of our patients on admission are listed in Table 1. Results obtained from the used questionnaires are presented in Table 2. Compared to the control group on first testing, we have found a statistically significant difference in all used tests and all groups of patients (*P* < .01). During the follow-up, we compared values obtained from QoL and QoS questionnaires in the control and patient group. The results are presented in Figures 1 and 2.

**Table 1:** Epidemiological characteristics of patients.

	Group I; N = 28	Group II; N = 47	Group III; N = 56
Age (year)	27.1 ± 2.4	45.1 ± 5.1	58.3 ± 4.4
Female (%)	39.4	53.5	52.6
Height (m)	1.73 ± 0.4	1.69 ± 0.3	1.70 ± 0.2
Weight (kg)	73.5 ± 11.5	84.0 ± 16.3	89.0 ± 18.3
Pain duration (months)	4.5 ± 0.5	5.8 ± 1.0	7.9 ± 2.3
Occupational status (%)			
Student	22.3	3.5	0
Permanent job	28.7	64.3	67.6
Occasional jobs	13.8	20.6	23.3
Unemployed	25.2	11.6	9.1
Type of job (%)			
Physical work	34.4	63.5	57.7
Intellectual work	65.6	36.5	42.3
Education (%)			
Primary studies	24.2	22.4	21.3
Secondary studies	42.1	50.2	58.3
University degree	33.7	27.4	20.4
Partner – yes (%)	27.5	61.3	76.4
Area of living (%)			
Town	85.3	77.3	68.5
Village	14.7	22.7	31.5

Group I: 18 to 20 yr, Group II: 30 to 50 yr, Group III: 50 to 65 yr.

**Table 2:** Scores on the obtained questionnaires.

		On admission	Month 6	Month 12	Month 24
ODI (0–100)	Gr I	18.3 ± 10.3	16.7 ± 8.3	15.1 ± 9.4*,†	13.5 ± 9.2*,†,‡
	Gr II	24.6 ± 12.7	23.7 ± 11.3	21.2 ± 9.2*,†	18.2 ± 9.8*,†,‡
	Gr III	26.8 ± 13.1	25.7 ± 12.5	24.8 ± 11.3	25.3 ± 9.7
VAS (0–10)	Gr I	6.4 ± 2.3	5.2 ± 2.0	4.3 ± 2.2*,†	2.3 ± 2.2*,†,‡
	Gr II	7.1 ± 2.7	6.2 ± 2.4	5.3 ± 2.6*,†	4.5 ± 2.3*,†,‡
	Gr III	7.3 ± 2.4	6.5 ± 1.9	5.5 ± 2.0	5.3 ± 2.2*,†
PDQ (0–38)	Gr I	16.5 ± 7.5	11.5 ± 8.0*,†	7.3 ± 3.3*,*,*,†,‡	4.0 ± 3.5*,*,*,†,‡,§
	Gr II	15.5 ± 6.3	11.2 ± 7.6*,†	7.5 ± 3.1*,*,†,‡	6.5 ± 3.3*,*,†,‡
	Gr III	15.2 ± 7.2	12.8 ± 8.3	8.2 ± 3.3*,*,†,‡	8.5 ± 3.5*,*,†,‡
SF-36 (0–100)	Gr I	65.4 ± 23.4	73.4 ± 24.0*,*,†	75.5 ± 21.7*,*,†	84.5 ± 25.9*,*,†,‡,§
	Gr II	57.4 ± 24.6	61.4 ± 24.6*,†	69.2 ± 22.7*,*,†,‡	72.2 ± 22.7*,*,*,†,‡,§
	Gr III	53.1 ± 23.8	54.4 ± 22.2	57.2 ± 22.2*,†	62.2 ± 23.1*,*,*,†,‡,§
PSQI (0–21)	Gr I	4.1 ± 2.3	3.2 ± 1.4*,†	2.9 ± 1.2*,*,†	2.8 ± 1.2*,*,†
	Gr II	5.8 ± 2.1	5.4 ± 1.4	4.6 ± 1.2*,†,‡	3.8 ± 1.2*,*,†,‡
	Gr III	7.2 ± 2.3	7.2 ± 1.9	6.9 ± 1.2	6.8 ± 1.2*,*,†
HARS (0–56)	Gr I	10.2 ± 5.3	8.9 ± 4.6	7.1 ± 3.1*,†,‡	4.3 ± 1.2*,*,*,†,‡,§
	Gr II	9.5 ± 6.2	8.8 ± 5.6	7.3 ± 5.1*,†,‡	5.8 ± 2.1*,†,‡
	Gr III	9.9 ± 6.2	8.9 ± 5.6	7.6 ± 5.1*,†	6.8 ± 2.1*,†,‡
HDRS (0–51)	Gr I	8.4 ± 5.4	7.6 ± 5.2	6.6 ± 5.2*,†	6.4 ± 5.0*,†
	Gr II	8.8 ± 5.6	7.7 ± 5.5	6.7 ± 4.8*,†	6.3 ± 5.0*,†
	Gr III	9.7 ± 6.2	8.3 ± 5.2	6.9 ± 5.4*,†	7.3 ± 5.0*,†

Group I: 18 to 20 yr, Group II: 30 to 50 yr, Group III: 50 to 65 yr.

HARS = Hamilton anxiety rating scale, HDRS = Hamilton depression rating scale, ODI = Oswestry low back pain disability index, PDQ = PainDETECT questionnaire, PSQI = the Pittsburg sleep inventory questionnaire, SF 36 = 36-item short-form survey instrument, VAS = 10-cm visual analogue scale.

† related to first testing.

‡ related to month 6.

§ related to month 12.

* *P* <.05.

** *P* <.01.

**Figure 3.**
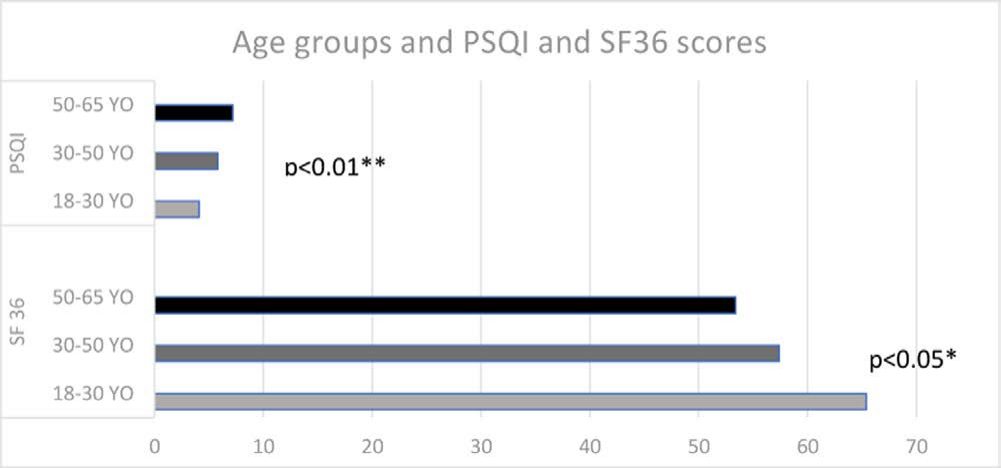
Differences in quality of life (SF36) and quality of sleep (PSQI) scores between different age groups based on first testing. PSQI = Pittsburgh sleep quality index

Based on the first assessment, between age groups, we did not find any statistically significant difference in severity (age was negatively associated with pain intensity, but with no statistical significance *P* > .05) or type of pain, anxiety, or depression. Elderly patients were more disabled (*P* < .01). In young individuals, SF36 scores were significantly higher (*P* < .05), and PSQI scores were lower (*P* < .01) than in the elderly (Fig. 3).

In our patients, worse QoL was associated with scores on ODI scale (rho = −0.56, *P* <.01), HARS (rho = −0.52, *P* <.01) HDRS (rho = −0.45, *P* <.05), visual analogue scale scale (rho = −0.42, *P* <.05), and especially the presence of neuropathic pain (rho = −0.63, *P* <.01). Worse sleep quality is associated with worse scores on pain questionnaires, especially with scores on the PDQ (rho = 0.58, *P* <.01). We did not find any statistically significant correlation with other epidemiological factors, but younger patients tend to have slightly worse scores on the used questionnaires. Neuropathic pain was an independent predictor of the lower SF36 scores – which indicates worse QoL (adjusted R^2^ = 0.73, *P* <.01 for the overall model) and higher PSQI scores – which indicates worse QoS (adjusted R^2^ = 0.71, *P* <.01 for the overall model).

## 4. Discussion

CLBP has become a global public health problem and poses a challenge, seriously affecting the QoL of patients. Psychosocial factors such as anxiety, and depression mainly contribute to the chronification of low back pain and disability.^[16]^ CLBP is a common condition that significantly influences patients’ workability and can cause higher anxiety and depression levels in these individuals. So, the relationship between psychosocial factors and CLBP is not simple. Due to these facts, besides medication therapy, alternative non-medication procedures such as cognitive behavioral therapy, clinical pilates, meditation, or acupuncture are proposed as complementary therapeutic procedures for accomplishing better outcomes.^[17,18]^

The patient’s QoL is related both to the influence of the disease and to the factors that are independent of the disease itself. Patient’s age is proposed as one of the factors accounting for interindividual differences in QoL among CLBP patients.^[19]^ In this study, we have focused on patients who are in the working population. This group is further divided into 3 subdivisions. We have found statistically significant differences between groups in QoL and QoS, which was of lower quality in groups of patients who were older than 50 (especially regarding sleep).

Coping with pain may get harder with advancing age due to additional age-associated decline in other functions, resulting in higher disability and lower QoL.^[20]^ Disability generally increases with advancing age, which is in conclusion with our findings.^[21]^ Previous studies with chronic pain patients suggest that pain intensity does not meaningfully vary between different age groups, which is supported by our results.^[22,23]^ On the other hand, well-being does not necessarily decline with advancing age.^[24]^ Previous studies were limited in that most of them focused on pain patients in general, without further differentiating between specific pain conditions such as CLBP. A study conducted by Wettstein et al on patients with CLBP found that mental health was better in older patients.^[19]^ Our study, based on our knowledge, is the first study focused on patients aged 18 to 65 exclusively. Our research is conducted on the working-age population, so the higher disability and inability to act following society’s role can be a reason for lower QoL in elderly groups of patients (for example, higher expectations of people who are of working age than the retired population).

Pain relief is the main index for evaluating the effects of treatment for CLBP patients.^[25]^ During the follow-up we noticed a reduction in pain in all groups and a reduction in disability scores in the population younger than 50. Reduction in pain scores is higher regarding neuropathic pain, which is probably influenced by the treatment. Treatment options for neuropathic pain are mainly drugs that have antidepressant and anxiolytic functions, so scores on HARS and DRS are also lower during the follow-up. As a disadvantage of our research, we can mention that we did not take into account the type and dosage of medication that were used, so we highly recommend that this needs to be taken into account in future studies regarding the QoL of patients with CLBP. Following previously mentioned, by the reduction of pain and disability, we noticed better QoL and QoS, especially in months 12 and 24, in comparison with scores on admission.

In some CLBP patients, there is a presence of a neuropathic pain component, and based on our research, this phenotype mainly influences worse QoL and QoS. In previous studies, neuropathic pain and QoL were significantly improved by antidepressants as compared to placebo groups, but anxiety was not significantly reduced.^[26]^ Also, the presence of neuropathic pain is connected with worse QoL.^[27]^ To our knowledge, unfortunately, the connection with lower QoS has not yet been questioned. Based on the mentioned, the identification of the main contributing pain mechanism, the presence of a neuropathic pain component are key in clinical management, which can significantly improve the QoL OF individuals with CLBP.

As a strength of our study, we can mention its longitudinal design. To our knowledge, this is the first study of QoL during a 2-year follow-up. We have tested the first subjects during the 6-month follow-up. period, but did not find statistically meaningful changes in scores on the obtained questionnaires, so we prolonged this period to 2 years. Of course, we encourage investigators to prolong this time period further in future studies. As a limitation of our study, we can mention that the participants were recruited from a tertiary care center; thus, results may not be representative of CLBP patients in general. Also, for a comprehensive assessment, a broader range of indicators needs to be included (as assessed by self-reports and by objective, performance-based tests as well as clinical assessment instruments). Our assessments were restricted only to self-report questionnaires. Also, the diagnosis of “neuropathic pain” or a “neuropathic component of pain,” is very complicated. In our study, it mainly depended on the PDQ score. A recent meta-analysis demonstrated that the PDQ’s sensitivity is 0.73 (95% CI = 0.56–0.84) and specificity is 0.81 (95% CI = 0.66–0.91). So, PDQ has weak recommendations for their use in the diagnostic pathway.^[28]^ There is no one reliable source for establishing the diagnosis of neuropathic pain, but in the future studies, the scores from patient reported outcomes can be associated with the clinical neurological examination, imaging techniques, and neurophysiologic tests, so the higher precision in establishing this diagnosis can be achieved. Also, correlation with different therapeutic measures needs to be taken into account in future studies.

Patients with severe pain and especially the presence of neuropathic pain, as well as those with anxiety and depression, have poorer QoL and QoS. Between age groups, patients older than 50 have lower QoL and QoS than young individuals. During the follow-up (two years), there was a reduction in pain, which was more pronounced in the groups of patients younger than 50. Our results encourage clinicians to observe and treat the neuropathic pain component (especially in the elderly) in patients with CLBP because it mainly influences QoL and QoS. Also, our results show that the immediate effect on QoL and QoS in these patients is very hard to establish, so prolonged treatment and observation of these patients is needed.

## Author contributions

**Conceptualization:** Aleksandar Stojanov, Srdjan Ljubisavljevic, Jelena Stojanov.

**Data curation:** Aleksandar Stojanov.

**Formal analysis:** Aleksandar Stojanov, Jelena Stojanov.

**Investigation:** Aleksandar Stojanov, Srdjan Ljubisavljevic, Jelena Stojanov.

**Methodology:** Srdjan Ljubisavljevic.

**Supervision:** Gordana Djordjevic, Biljana Zivadinovic.

**Writing – original draft:** Aleksandar Stojanov.

**Writing – review & editing:** Gordana Djordjevic, Biljana Zivadinovic.
